# Case Report: Contrast-enhanced ultrasonography for evaluating a newly detected contralateral pulmonary lesion during non-small cell lung cancer chemoimmunotherapy

**DOI:** 10.3389/fimmu.2026.1838122

**Published:** 2026-05-21

**Authors:** Yang Liu, Lusi Feng, Xiaoqian Yang, Shiyu Wen, Yu Xiang, Xuelei Ma, Rongxing Zhou, Wei Du

**Affiliations:** 1 Day Surgery Center, General Practice Medical Center, West China Hospital, Sichuan University, Chengdu, Sichuan, China; 2 Department of Biotherapy, Cancer Center and State Key Laboratory of Biotherapy, West China Hospital, Sichuan University, Chengdu, Sichuan, China; 3 Division of Biliary Surgery, Department of General Surgery, West China Hospital, Sichuan University, Chengdu, Sichuan, China

**Keywords:** CEUS, immune checkpoint inhibitors, NSCLC, pembrolizumab, peripheral pulmonary lesion, pulmonary infection

## Abstract

**Background:**

New pulmonary lesions arising during chemoimmunotherapy for non-small cell lung cancer (NSCLC) pose a common diagnostic dilemma because they may reflect tumor progression, infection, treatment-related inflammation, or other benign processes. Computed tomography remains central to surveillance, but morphology alone is sometimes insufficient for confident characterization. Contrast-enhanced ultrasonography (CEUS) can provide real-time perfusion information in peripheral pulmonary lesions and may offer incremental value in selected subpleural lesions.

**Case presentation:**

A 78-year-old man with stage IIIB squamous NSCLC received four cycles of paclitaxel plus cisplatin combined with pembrolizumab. During treatment, he developed a newly detected contralateral lesion in the posterior basal segment of the left lower lobe. Transthoracic lung ultrasonography demonstrated a subpleural hypoechoic lesion measuring approximately 2.4 × 2.2 cm. CEUS showed rapid hyperenhancement in the pulmonary arterial phase, relatively homogeneous internal enhancement, and slight persistent hyperenhancement in the venous phase, favoring an inflammatory rather than a malignant lesion. This interpretation was supported by contemporaneous microbiologic evidence of respiratory infection and was subsequently confirmed clinically by marked lesion regression on short-interval follow-up computed tomography after anti-infective treatment.

**Conclusion:**

This case highlights the potential adjunctive role of CEUS in the evaluation of newly detected peripheral pulmonary lesions during chemoimmunotherapy. Current evidence indicates that CEUS is most useful when integrated with clinical, laboratory, microbiologic, and cross-sectional imaging data, rather than used as a stand-alone discriminator between benign and malignant disease.

## 1 Introduction

Immune checkpoint inhibitors, administered alone or in combination with platinum-based chemotherapy, are now integral to the treatment of advanced non-small cell lung cancer (NSCLC) without actionable driver alterations (1). As systemic treatment options have evolved, interpretation of interval thoracic imaging has become more complex. A newly detected pulmonary lesion during therapy may indicate true tumor progression, superimposed infection, immune-related inflammatory toxicity, or another non-malignant process (2, 3). In older patients with recent hospitalization, pleural intervention, or concurrent respiratory symptoms, computed tomography (CT) morphology alone may not always allow confident discrimination among these possibilities.

Lung ultrasonography is increasingly recognized as a useful bedside tool for evaluating pleural and subpleural abnormalities, and contrast-enhanced ultrasonography (CEUS) can further depict lesion perfusion in real time (4, 5). In peripheral pulmonary lesions, CEUS has been explored as an adjunctive technique for lesion characterization, differential diagnosis, and biopsy planning (6, 7). Available reviews and pooled analyses suggest that CEUS may be most informative when lesions are pleural- based or immediately subpleural and therefore technically accessible to transthoracic assessment (4–6).

Here, we report the case of an elderly man with stage IIIB squamous NSCLC in whom CEUS contributed materially to the interpretation of a newly detected contralateral pulmonary lesion during pembrolizumab-based chemoimmunotherapy. To place the diagnostic reasoning in context, we also discuss the application of CEUS in the differential diagnosis of peripheral pulmonary lesions, with particular emphasis on the distinction between inflammatory and malignant subpleural lesions.

## 2 Case presentation

**Figure 1:**
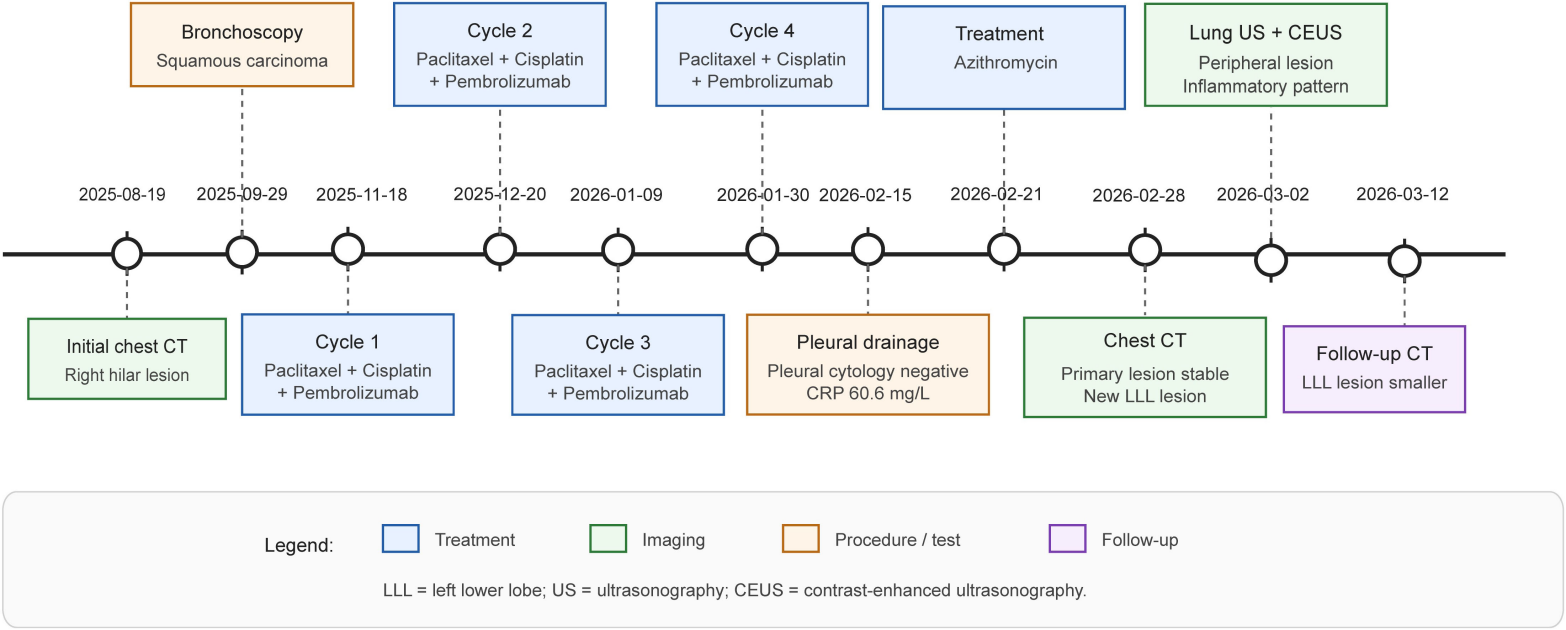
Timeline of the clinical course. Key events included initial detection of the right hilar lesion on August 19, 2025; bronchoscopic confirmation of squamous-cell carcinoma on September 29, 2025; four cycles of paclitaxel-cisplatin plus pembrolizumab on November 18, 2025, December 20, 2025, January 9, 2026, and January 30, 2026; pleural drainage in February 15, 2026; anti-infective treatment in February 15, 2026; detection of a new lesion in the left lower lobe on February 28, 2026; CEUS on March 2, 2026; microbiologic reassessment and modified chemotherapy on March 3, 2026; and follow-up CT showing marked shrinkage of the left lower lobe lesion on March 12, 2026.

A 78-year-old man was admitted because of hemoptysis for more than 6 months, a diagnosis of squamous-cell lung carcinoma 4 months earlier, and a productive cough with yellow sputum for 6 days. The major diagnostic and therapeutic events are summarized in **Figure 1**.

Approximately 6 months before admission, he began to have intermittent hemoptysis, consisting of small amounts of bright-red blood mixed with sputum. The symptoms improved after hemostatic therapy. On August 19, 2025, chest CT at our hospital showed a soft-tissue opacity near the hilar region of the right lower lung, with surrounding obstructive inflammation; right hilar and mediastinal lymphadenopathy was also present. Systemic staging, including body CT and bone scintigraphy, showed no brain, abdominal, or osseous metastases.

On September 29, 2025, bronchoscopy with biopsy was performed. Histopathological examination showed squamous-cell carcinoma. Immunohistochemical analysis showed a PD-L1 tumor proportion score of approximately 2%. Next-generation sequencing identified missense alterations in TP53, ALK, and CDKN2A. The clinical stage was assessed as cT4N2bM0, stage IIIB.

After multidisciplinary discussion, the patient began systemic therapy with paclitaxel plus cisplatin combined with pembrolizumab, which was consistent with contemporary treatment strategies for advanced NSCLC (1). Four cycles were administered on November 18, 2025; December 20, 2025; January 9, 2026; and January 30, 2026.

On February 15, 2026, he was admitted to another hospital because of upper-extremity swelling. Ultrasonography revealed a large pleural effusion, and thoracentesis with drainage was performed using a small-bore intercostal catheter (6–10 Fr). Cytologic examination of the pleural fluid showed no malignant cells. No pleural fluid biochemical analysis was available; this was a limitation of the outside-hospital evaluation, and standard biochemical testing (protein, lactate dehydrogenase, pH, and glucose) was not performed at that institution. At that time, laboratory testing showed a C-reactive protein level of 60.6 mg/L, a red-cell count of 3.56 × 10^12/L, a hemoglobin level of 111 g/L, a hematocrit of 0.34 L/L, a platelet count of 127 × 10^9/L, and a white-cell count of 6.22 × 10^9/L. The neutrophil percentage was 72.4%, the lymphocyte percentage was 15.9%, and the monocyte percentage was 10.8%. He improved after pleural drainage and anti-infective treatment.

On February 21, 2026, he developed a cough productive of yellow sputum. Sputum culture grew Moraxella catarrhalis with beta-lactamase positivity; the isolate was susceptible to trimethoprim-sulfamethoxazole, tetracycline, erythromycin, amoxicillin-clavulanate, and azithromycin. Intravenous azithromycin was started, with partial symptomatic improvement. Following discharge from the external hospital, he was referred to our institution for further evaluation of persistent respiratory symptoms and a newly identified pulmonary lesion.

**Figure 2:**
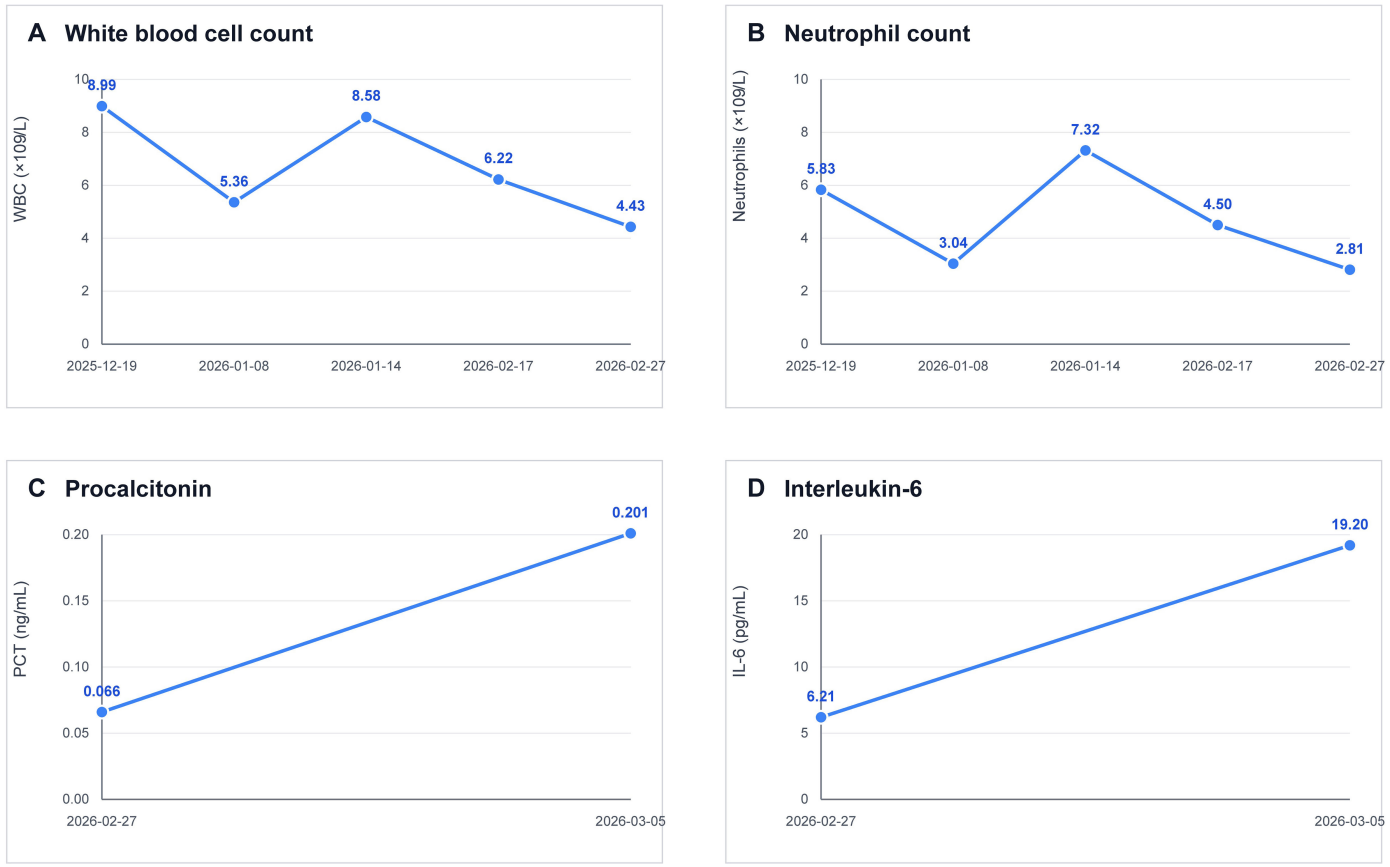
Serial laboratory trends across the clinical course. **(A)** White blood cell count from December 19, 2025, to February 27, 2026. **(B)** Absolute neutrophil count from December 19, 2025, to February 27, 2026. **(C)** Procalcitonin level from February 27, 2026, to March 5, 2026. **(D)** Interleukin-6 level from February 27, 2026, to March 5, 2026.

On February 27, 2026, laboratory testing at our hospital showed a hemoglobin level of 114 g/L, a platelet count of 43 × 10^9/L, a white-cell count of 4.23 × 10^9/L, and an absolute neutrophil count of 2.81 × 10^9/L. The procalcitonin level was 0.066 ng/mL. Body temperature remained within the normal range throughout the observation period, and no obvious fever was documented. Serial laboratory testing showed dynamic changes in inflammatory and hematologic markers across the observation period. The white blood cell count decreased from 8.99 × 10^9/L on December 19, 2025, to 4.43 × 10^9/L on February 27, 2026, and the absolute neutrophil count decreased from 5.83 × 10^9/L to 2.81 × 10^9/L over the same interval, reflecting trends during chemotherapy and early inflammatory changes. In contrast, the procalcitonin level increased from 0.066 ng/mL on February 27, 2026, to 0.201 ng/mL on March 5, 2026, and interleukin-6 increased from 6.21 pg/mL to 19.20 pg/mL, which characterize the acute infectious episode. These serial changes are summarized in **Figure 2**. Thyroid-function testing showed marked hypothyroidism, with a thyroid-stimulating hormone level of 47.700 mIU/L, a total triiodothyronine level of 0.32 nmol/L, a free triiodothyronine level of 0.71 pmol/L, and a total thyroxine level of 12.40 nmol/L. Levothyroxine was started after endocrine consultation.

Venous ultrasonography on February 27, 2026, showed thickening of the wall of the right common femoral vein, raising concern for phlebitis; reflux in both common femoral veins; and small-caliber cephalic veins in both upper arms. Transthoracic echocardiography on February 28, 2026, showed masses near the roof of both atria, slight left atrial enlargement, and preserved left ventricular systolic function. Magnetic resonance imaging of the brain showed no metastases. CT of the abdomen showed a probable hemangioma in the right anterior hepatic lobe, a fatty lesion at the lower pole of the left kidney, bilateral adrenal nodular thickening suggestive of hyperplasia or adenoma, and several high-density nodules in the lumbosacral vertebrae that were considered likely bone islands.

**Figure 3:**
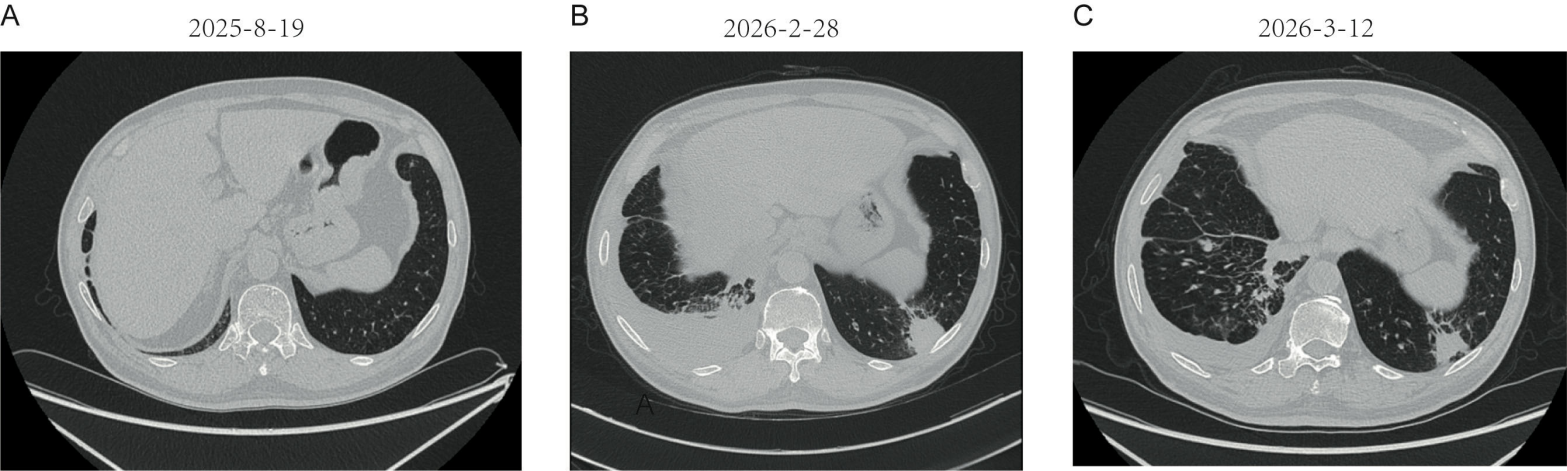
Chest CT findings. **(A)** No soft-tissue lesion was detected in the posterior basal segment of the left lower lobe on August 19, 2025. **(B)** A newly detected soft-tissue lesion was seen in the posterior basal segment of the left lower lobe on February 28, 2026. **(C)** Marked shrinkage of the left lower lobe lesion was observed on follow-up CT obtained on March 12, 2026.

Chest CT on February 28, 2026, showed that the irregular soft-tissue lesion near the right hilum in the right middle and lower lung was essentially unchanged from the scan of February 17, 2026. Bilateral pleural thickening was present, nodular in places, with a right pleural effusion and possible pleural metastases. Most notably, a new soft-tissue lesion had appeared in the posterior basal segment of the left lower lobe. The radiologic differential diagnosis included inflammatory disease and metastasis. Representative CT images of the newly detected left lower lobe lesion and its interval regression are shown in **Figure 3**.

**Figure 4:**
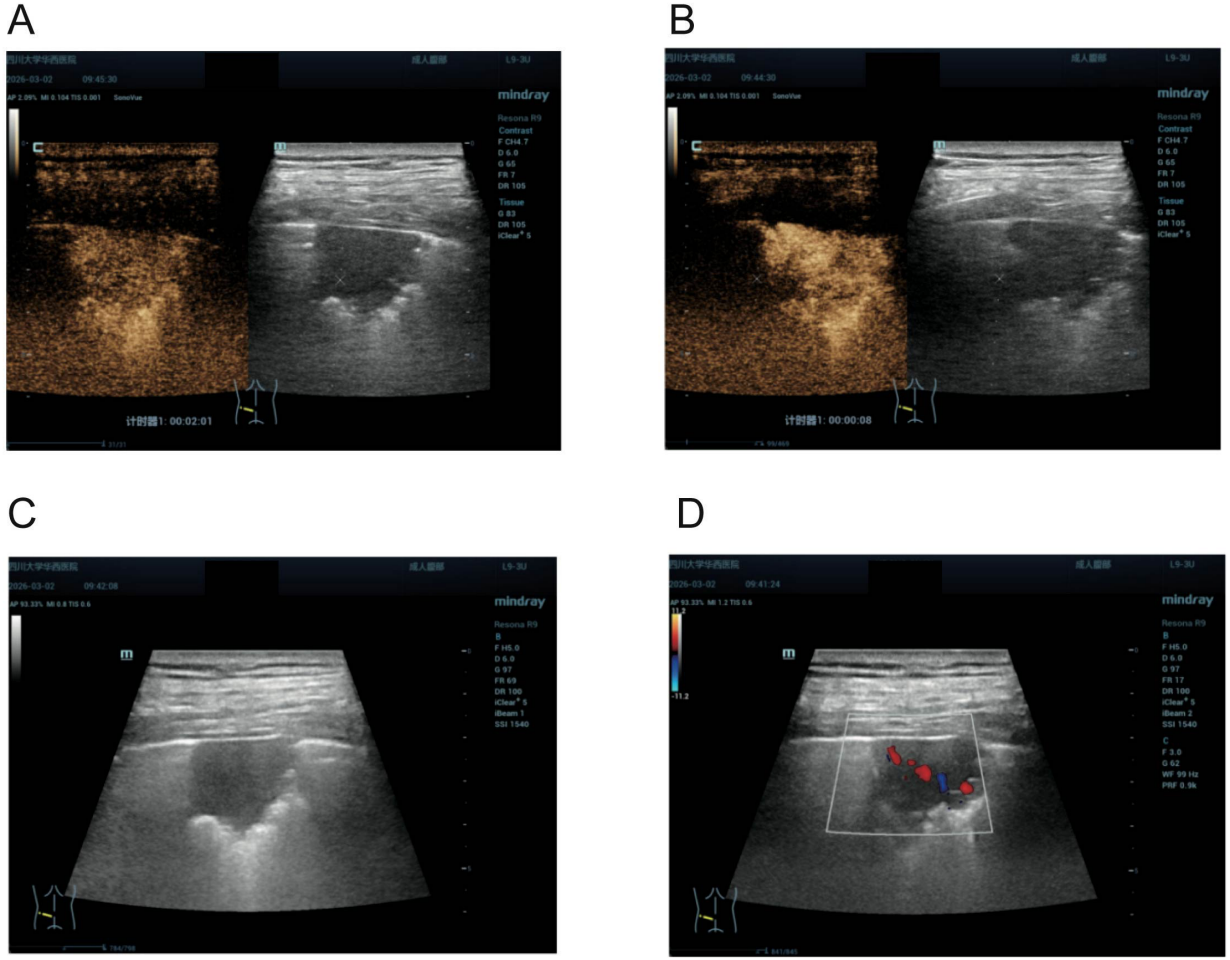
Multimodal ultrasonographic findings of the left lower lobe lesion. **(A)** CEUS shows relatively homogeneous internal enhancement with slight persistent hyperenhancement in the venous phase, favoring an inflammatory lesion. **(B)** CEUS shows hyperenhancement in the pulmonary arterial phase. **(C)** Gray-scale ultrasonography shows a hypoechoic subpleural lesion in the dorsal left lower lobe, measuring approximately 2.4 × 2.2 cm. **(D)** Color Doppler imaging shows marked intralesional vascularity, with obvious blood-flow signals detected within the lesion.

Because the new lesion was peripheral and amenable to sonographic assessment, transthoracic lung ultrasonography and CEUS were performed on March 2, 2026. Gray-scale ultrasonography showed a hypoechoic lesion in the dorsal left lower lobe measuring approximately 2.4 × 2.2 cm, with an indistinct deep margin. Color Doppler imaging demonstrated obvious intralesional blood-flow signals. CEUS showed hyperenhancement during the pulmonary arterial phase, relatively homogeneous internal enhancement, and slight persistent hyperenhancement during the venous phase. Quantitative parameters were as follows: arrival time (AT) = 5.58 s, time-to-peak (TTP) = 23.58 s, peak intensity (PI) = 23.6 dB, wash-in slope (WIS) = 4.40, and wash-out time (WOT) = 256 s. The short AT and TTP, steep WIS, and prolonged WOT were consistent with an inflammatory rather than a malignant perfusion pattern. Gray-scale ultrasonography and CEUS findings are shown in **Figure 4**.

A multidisciplinary conference involving specialists in infectious diseases, pulmonology, and radiology was convened. The consensus was that the new lesion in the left lower lobe was more likely inflammatory than malignant, in view of the patient’s purulent sputum, documented *M. catarrhalis* infection, the stability of the known primary lesion, and the perfusion pattern on CEUS. Further microbiologic studies were recommended, Pembrolizumab was temporarily withheld in view of the active respiratory infection, as continuing immunotherapy during uncontrolled infection may worsen infectious control, increase complication risk, and complicate subsequent clinical assessment. Anti-infective treatment was continued, and interval chest imaging was planned. Bronchoscopy or percutaneous biopsy was reserved in the event the lesion failed to regress.

On March 3, 2026, additional testing for Mycoplasma pneumoniae IgM, Chlamydia pneumoniae IgM, fungal β-D-glucan, galactomannan, cryptococcal antigen, tuberculosis interferon-γ release assay, purified protein derivative skin test, sputum smear, and repeat sputum culture was negative. Aspergillus IgG was positive at greater than 120 AU/mL. After review by infectious-disease consultants, expectorant therapy and serial monitoring of the blood count and fungal markers were recommended.

Follow-up chest CT on March 12, 2026, showed marked shrinkage of the soft-tissue lesion in the left lower lobe. No biopsy of that lesion was performed. Pleuroscopy was not performed at the initial stage; this decision reflected a combination of factors, including the patient’s advanced age, the favorable response to anti-infective treatment, the CEUS findings favoring an inflammatory etiology, and the multidisciplinary consensus to pursue a conservative watch-and-wait approach before resorting to invasive procedures. However, the interval decrease in size, together with the clinical context and CEUS findings, strongly supported the conclusion that the lesion was inflammatory rather than metastatic.

## 3 Discussion

This case highlights a recurring problem in thoracic oncology: how should a newly detected peripheral pulmonary lesion be interpreted in a patient who is already receiving chemoimmunotherapy for known lung cancer? In routine practice, the immediate concern is often disease progression. However, in immunotherapy-treated patients, the differential diagnosis is wider and includes bacterial infection, immune-related pneumonitis, treatment-associated inflammatory change, infarction, and other benign processes (2, 3, 8–12). The key clinical challenge is therefore not simply lesion detection, but timely characterization that is sufficiently reliable to guide management while avoiding both overtreatment and unnecessary delay. A structured overview of the key differentiating features between tumor progression and infectious/inflammatory lesions, including CEUS perfusion characteristics, CT morphology, and clinical/laboratory findings, is provided in **Supplementary Table 1**.

In the present patient, several features argued against immediate classification of the new lesion as metastatic progression. The known right-sided primary lesion was essentially stable, the newly detected left lower lobe lesion was peripheral and technically accessible to ultrasound, microbiologic testing documented Moraxella catarrhalis infection, inflammatory biomarkers changed dynamically over time, and the lesion subsequently regressed after anti-infective treatment. Within this broader clinical context, CEUS provided useful additional information by showing rapid and relatively homogeneous enhancement without a convincing necrotic non-enhancing core, a pattern that favored inflammatory change over malignant progression.

The available literature suggests that the role of CEUS in pulmonary disease is narrow but potentially valuable. Thoracic CEUS is most relevant when the lesion is pleural-based or immediately subpleural and thus accessible to transthoracic examination. Reviews from Radiographics and the World Federation for Ultrasound in Medicine and Biology have emphasized that CEUS can depict microvascular perfusion in real time, improve delineation of viable versus necrotic tissue, and complement baseline gray-scale ultrasound in selected peripheral lung lesions (4, 5). Importantly, CEUS contrast agents are administered in small volumes, carry no nephrotoxic risk, and can be safely used in patients with renal impairment or end-stage renal disease, making CEUS a particularly attractive option in elderly oncologic patients who may not tolerate iodinated contrast-enhanced CT. A 2024 systematic review and meta-analysis focusing on benign versus malignant subpleural lung lesions concluded that CEUS has good overall diagnostic performance, while also emphasizing substantial heterogeneity in patient selection, acquisition protocols, reference standards, and contrastographic criteria across studies (6). A subsequent systematic review of quantitative CEUS reached a similar conclusion: no single perfusion variable is universally reliable, whereas combined or model-based approaches appear more robust than any isolated sign (13).

The original studies in this field can be broadly organized into three themes. First, early feasibility and observational studies established that CEUS could be applied to peripheral pulmonary lesions. For instance, Sperandeo et al. (n = 98) (14) and Caremani et al. (n = 60) (15) described distinct enhancement patterns in infectious versus neoplastic lesions, while Quaia et al. (16) confirmed the technical feasibility of low-mechanical-index CEUS for thoracic characterization. However, these foundational reports also underscored substantial overlap between inflammatory and malignant lesions, indicating their adjunctive rather than stand-alone value. Second, later investigations focused on time-based quantitative parameters. Tang et al. (n = 96) proposed the time difference of arrival (TDOA) to distinguish benign inflammatory lesions from malignant peripheral pulmonary lesions and reported encouraging diagnostic performance (17). Bi et al. refined this concept by introducing the contrast-agent arrival-time difference ratio, which improved benign-malignant discrimination in subpleural lesions, though noting that interpretation remains context-dependent (18). Shen et al. similarly reported that enhancement characteristics and necrosis-related findings were useful adjuncts, particularly when combined with baseline sonographic findings (19). Third, more recent multiparametric studies and long-term clinical experiences, such as the 5-year retrospective analysis by Li et al. (7), suggest that CEUS performs best when interpreted together with conventional ultrasound features and the broader clinical context. Its incremental value is most evident in lesion characterization and biopsy planning by identifying viable enhancing tissue (7, 20).

Against this background, the present case should be viewed not as proof of diagnostic certainty from CEUS alone, but as an example of how CEUS may contribute to probability refinement within a multimodal assessment. In our patient, the interpretation in favor of inflammation was based on the combined weight of the CEUS pattern, the absence of obvious internal non-enhancing necrosis, microbiologic evidence of respiratory infection, the stability of the known primary tumor, and short-interval regression of the contralateral lesion. This integrative approach is consistent with the current literature, which supports CEUS as a contextual decision-support tool rather than a stand-alone rule-in or rule-out test for malignancy.

Equally important, the literature defines the limitations of pulmonary CEUS. Sperandeo et al. reported that CEUS did not reliably discriminate community-acquired pneumonia from lung cancer when used in isolation (21). More recent analyses likewise caution that contrast-arrival time alone may not provide dependable benign-versus-malignant classification across heterogeneous peripheral pulmonary lesions, given the dual vascular supply of the lung, variable inflammatory activity, lesion necrosis, and histopathologic diversity (6, 13, 21). This caution is particularly relevant in patients receiving immunotherapy, because infection, treatment-related pneumonitis, and tumor progression may all manifest as new or changing pulmonary opacities (2, 3, 8–12).

Another important branch of the literature concerns procedural guidance. Studies of CEUS in peripheral pulmonary lesions have shown that it can improve biopsy targeting by identifying viable enhancing tissue and helping operators avoid necrotic components (5, 7, 20). Meta-analytic evidence further suggests that CEUS-guided biopsy may improve diagnostic yield in selected thoracic lesions without materially increasing complications (13). Although biopsy of the new lesion was ultimately unnecessary in our patient because the lesion regressed promptly, CEUS still influenced management in a clinically meaningful way: it supported a conservative strategy of temporary immunotherapy interruption, continuation of anti-infective therapy, and short-interval radiologic reassessment rather than immediate oncologic escalation or urgent invasive sampling.

The principal clinical message of this case is pragmatic. In patients with lung cancer receiving chemoimmunotherapy, a newly detected contralateral peripheral pulmonary lesion should not automatically be interpreted as progressive disease. When the lesion is subpleural and technically accessible, CEUS may provide incremental information that is particularly useful when the dominant differential diagnosis is inflammation versus malignant progression. The value of CEUS appears greatest when it is embedded in multidisciplinary reasoning that integrates symptoms, laboratory markers, microbiology, CT findings, and interval imaging response (2–6, 13, 17–21).

Several limitations should be acknowledged. First, the left lower lobe lesion was not pathologically confirmed, and the final diagnosis, therefore, remained clinicoradiologic rather than histologic. Second, the specific microbial etiology of the left lower lobe lesion remains presumptive, as *M. catarrhalis* was isolated from sputum rather than directly from the lesion itself. Third, this is a single case report, and its implications should not be generalized beyond carefully selected peripheral lesions. Fourth, the pulmonary CEUS literature remains heterogeneous with respect to patient selection, acquisition protocols, perfusion definitions, and reference standards (6, 13). Nevertheless, the convergence of microbiologic evidence, inflammatory biomarker trends, CEUS findings, multidisciplinary assessment, and rapid lesion regression strongly supports an inflammatory etiology in this patient.

## 4 Conclusion

In conclusion, this case report suggests that CEUS can serve as a useful adjunct in the evaluation of newly detected peripheral pulmonary lesions arising during chemoimmunotherapy for NSCLC. The current evidence does not support the use of CEUS as an isolated diagnostic test for malignancy. Rather, its main value lies in refining pretest probability, supporting biopsy planning when needed, and helping clinicians avoid premature attribution of every new pulmonary lesion to tumor progression.

## Data Availability

The original contributions presented in the study are included in the article/**Supplementary Material**. Further inquiries can be directed to the corresponding author.
