# Supplementary material for: Case Report: Contrast-enhanced ultrasonography for evaluating a newly detected contralateral pulmonary lesion during non-small cell lung cancer chemoimmunotherapy
Source: Front Immunol. 2026 May 21;17:1838122. doi: 10.3389/fimmu.2026.1838122 (PMC13233419; doi:10.3389/fimmu.2026.1838122)
Supplement: Supplementary file 1 [file Table1.docx]

Supplementary Material

# Supplementary Table S1. Comparative features of tumor progression versus infectious/inflammatory lesions in peripheral pulmonary lesions during chemoimmunotherapy.

| **Feature** | **Category** | **Tumor Progression** | **Infectious/Inflammatory Lesion** |
| --- | --- | --- | --- |
| Enhancement pattern | CEUS | Heterogeneous, irregular | Homogeneous, uniform |
| Enhancement timing | CEUS | Delayed or slow wash-in | Rapid arterial-phase hyperenhancement |
| Wash-out pattern | CEUS | Early wash-out | Prolonged or persistent enhancement |
| Internal necrosis | CEUS | Non-enhancing necrotic core common | Absent or minimal |
| Lesion morphology | CT | Irregular margins, spiculation | Consolidation, ground-glass opacity, air bronchogram |
| Primary lesion status | CT | Enlarging or new lesions | Stable |
| Treatment response on imaging | CT | Progressive enlargement | Regression after anti-infective therapy |
| Symptoms | Clinical | Non-specific, indolent onset | Productive cough, purulent sputum |
| Inflammatory markers | Clinical | Normal or mildly elevated | CRP, PCT, IL-6 elevated with dynamic changes |
| Microbiology | Clinical | Negative | Positive culture or serology |
| Response to antibiotics | Clinical | No regression | Marked lesion regression |

**Abbreviations:** CEUS, contrast-enhanced ultrasonography; CT, computed tomography; CRP, C-reactive protein; PCT, procalcitonin; IL-6, interleukin-6
